# Future Perspectives in Senescence-Based Therapies for Head and Neck Cancer

**DOI:** 10.3390/cancers17121965

**Published:** 2025-06-12

**Authors:** Bruna Haddad Palomares, Manoela Domingues Martins, Marco Antonio Trevizani Martins, Cristiane Helena Squarize, Rogerio Moraes Castilho

**Affiliations:** 1 Oral Diagnosis Department, Piracicaba School of Dentistry, State University of Campinas, Piracicaba 13414-903, SP, Brazil; brunahddpalomares@gmail.com; 2 Department of Oral Pathology, School of Dentistry, Universidade Federal do Rio Grande do Sul, Porto Alegre 90035-003, RS, Brazil; manomartins@gmail.com (M.D.M.); kekomartins@yahoo.com.br (M.A.T.M.); 3 Department of Oral Medicine, Hospital de Clínicas de Porto Alegre, Porto Alegre 90035-903, RS, Brazil; 4 Laboratory of Epithelial Biology, Department of Periodontics and Oral Medicine, School of Dentistry, University of Michigan, Ann Arbor, MI 48109, USA; csquariz@umich.edu; 5 Rogel Cancer Center, University of Michigan, Ann Arbor, MI 48109, USA

**Keywords:** chromatin organization, cellular senescence, therapy-induced senescence, epigenetic regulation, biomarkers, cancer therapy, HNSCC

## Abstract

Cellular senescence is a complex physiological process in which cells permanently stop dividing and enter a stable state of cell-cycle arrest. This mechanism is typically triggered by various stressors, such as DNA damage, oxidative stress, telomere shortening, and oncogene activation. Senescent cells remain metabolically active and significantly influence their microenvironment through the senescence-associated secretory phenotype (SASP), which includes the secretion of inflammatory cytokines, growth factors, and proteases. While cellular senescence serves as a crucial tumor-suppressive mechanism by preventing the proliferation of damaged or potentially cancerous cells, it also plays a paradoxical role by promoting chronic inflammation, tissue dysfunction, and potentially oncogenesis. Therefore, understanding the regulation and impact of cellular senescence is vital for developing therapeutic interventions that leverage its benefits while minimizing adverse outcomes. In this review, we provide an overview of the current understanding of cellular senescence in cancer biology and discuss the emerging field of senescence-targeted therapies. We focus specifically on the role of senescence in head and neck cancers, examining the potential of induced senescence therapy to mitigate the progression of these tumors. This review aims to correlate the dual nature of senescence with innovative therapeutic strategies, highlighting its promise and challenges in improving treatment outcomes for HNC patients.

## Simple Summary

Head and neck cancers present significant therapeutic challenges due to high rates of recurrence and limited treatment success. This review examines cellular senescence, a state in which damaged cells cease to divide, as a mechanism that can both suppress and promote tumor progression. The authors discuss emerging therapeutic strategies that induce senescence in cancer cells, alongside approaches to eliminate these cells to prevent adverse effects. By integrating senescence-inducing agents with immune modulation and targeted therapies, this study highlights a promising direction for improving patient outcomes and reducing relapse in head and neck cancer management.

## 1. Introduction

Head and neck cancers (HNCs) represent a prevalent and often lethal group of malignancies originating not only from the mucosal linings of the oral cavity, pharynx, and larynx but also from the paranasal sinuses and salivary glands. Squamous-cell carcinoma, in particular, is the sixth most common non-skin cancer worldwide. Traditionally, tobacco use and alcohol consumption have been recognized as major etiological factors; however, infection with high-risk human papillomavirus (HPV) has emerged as a significant contributor in a distinct subset of cases, particularly in oropharyngeal tumors, where it is associated with lower overall mutation rates and unique prognostic profiles [1]. Recent genomic studies, including whole-exome sequencing of tumor–normal pairs, have shed light on the intricate mutational landscape of HNC. These investigations have revealed a high load of coding mutations affecting established cancer genes such as *TP53*, *CDKN2A*, *PTEN*, *PIK3CA*, and *HRAS*, as well as novel alterations in genes that regulate squamous differentiation (e.g., *NOTCH1*, *IRF6*, and *TP63*) [2]. Moreover, widespread epigenetic modification, including abnormal DNA methylation, histone modifications, altered chromatin remodeling, and changes in non-coding RNA expression, further complicates this molecular landscape [3]. Collectively, these genetic and epigenetic alterations drive carcinogenesis, tumor progression, and resistance to therapy by promoting processes such as the emergence of cancer stem cells, underscoring the complexity of head and neck cancers [3,4].

Given these challenges, researchers are exploring innovative therapeutic avenues that harness intrinsic cellular mechanisms to suppress tumor progression. Cellular senescence, a state of stable and irreversible cell-cycle arrest triggered by stressors such as DNA damage, telomere shortening, or oncogene activation, serves as a critical tumor-suppressive barrier by halting the proliferation of damaged cells [5,6,7]. Unlike apoptosis, senescence preserves cells in a metabolically active state, enabling them to influence the tumor microenvironment through the senescence-associated secretory phenotype (SASP) [8].

Therapeutic induction of senescence has emerged as a promising strategy to suppress tumor growth and overcome treatment resistance. Epigenetic regulators play a central role in controlling senescence. For example, histone deacetylase inhibitors (HDACi) such as Trichostatin A (TSA) and SAHA trigger hyperacetylation of histones, which has been shown to reduce cancer stem cell populations and sensitize HNSCC cells to chemotherapeutic agents like cisplatin [3]. Unlike other emerging treatments, senescence-based therapies uniquely target both proliferating tumor cells and cancer stem cells while reshaping the tumor microenvironment.

Future directions in senescence therapy include the development of senolytic agents that selectively eliminate senescent cells, as well as combination approaches that integrate senescence with apoptosis pathways to enhance therapeutic efficacy [8,9,10]. In addition, immunotherapeutic strategies that eliminate senescent cells via immune checkpoint modulation, such as PD-1 and CTLA-4 blockade, represent another promising avenue [11]. This review highlights emerging opportunities in induced senescence therapy for HNC, focusing on novel targets, biomarkers, and pharmacological agents. It also presents the latest preclinical and clinical studies pertinent to senescence therapy for HNC. By understanding the delicate balance between the tumor-suppressive and tumor-promoting roles of senescence, the researchers aim to develop new therapeutic strategies that improve treatment outcomes and long-term prognosis for patients with HNC.

## 2. Senescence

### 2.1. Dual Nature

During development, cells transition through various phenotypic states, each characterized by specific gene expression patterns, protein–protein interactions, and metabolic reactions. Programmed cell death plays a critical role by removing unwanted cells during embryonic development [12]. Similarly, cellular senescence is observed in several tissues, including the developing limbs, nervous system, and gut endoderm, playing a crucial role in clearing out cells that are no longer needed [13]. This is the case of developing kidneys, in which senescent cells trigger a macrophage phagocytic response, leading to the regression of the mesonephros [14].

Similarly observed during development, somatic cells can enter a state of cellular senescence defined by cell-cycle arrest. This state can be triggered by various challenges, such as DNA damage, oncogene activation, and telomere shortening. The gradual reduction in telomere length limits the replicative capacity of somatic cells, culminating in cellular senescence, a phenomenon known as the Hayflick limit. Unlike apoptosis, senescence involves living cells with high metabolic activity that communicate with neighboring cells and the immune system via the SASP [8].

**Figure 1:**
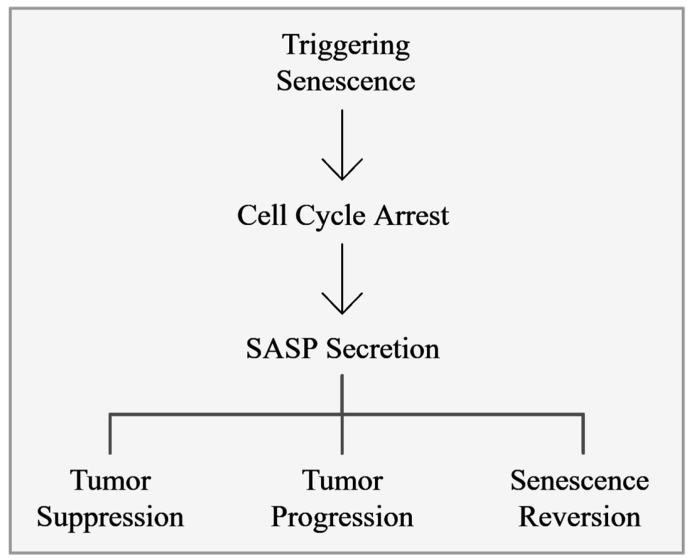
Schematic illustration of the nature of cellular senescence in cancer: Cellular senescence suppresses tumor growth by arresting proliferation, yet persistent senescent cells can drive cancer progression via chronic SASP secretion and rare escape mechanisms like EMT or neosis [22,23]. This figure was generated using Napkin AI.

From a cancer perspective, cellular senescence functions as a physiological mechanism to suppress tumor development by enforcing a permanent stop in cell division, inducing resistance to apoptotic stimuli, causing morphologic alterations, and deregulating metabolism, effectively curbing the unchecked growth of potential malignancies [15,16]. Nonetheless, senescence also plays a paradoxical role in cancer progression, in which the presence of persistent senescent cells in the tumor contributes to oncogenesis through several mechanisms, including the SASP (Figure 1) [8,17,18,19]. The puzzling role of cellular senescence in cancer is explained by Moiseeva et al., who categorize senescence into acute and chronic types. Acute senescence serves as an adaptive mechanism in response to stress, contributing significantly to wound healing and the suppression of tumors. During this process, senescent cells stimulate the immune system to remove them and assist in tissue repair, which provides a longer-lasting and more effective signaling function compared to apoptosis [20,21]. On the other hand, chronic senescence involves ongoing inflammation that can drive age-associated diseases, including cancer, by enhancing tumor growth, migration, invasiveness, formation of new blood vessels, and epithelial–mesenchymal transition (EMT) [20]. Studies have also indicated that senescence reversion might occur through gene cocktails inducing pluripotent stem cells or EMT transcription factors like Twist 1 or Twist 2. However, it remains unclear whether this reversion is due to an active escape process or the resumption of proliferation in partially senescent cells [20]. Moreover, polyploid cells may be able to escape from senescence via a process called de-polyploidization or neosis [22,23], resulting in aggressive and chemoresistant mitotically active clones [24]. These cells, by secreting factors like VEGF and MIF, promote the survival and progression of neighboring cancer cells, although this escape is rare [25].

### 2.2. Characteristics and Biomarkers

Senescent cells exhibit distinct morphological features, such as a flattened shape and enlarged nuclei, that arise from decreased proteasome peptidase activity, leading to protein accumulation and changes in intracellular lipid composition affecting the mitochondria and lysosomes [15,26]. Based on the time elapsed since induction and the reversibility of the phenotype, senescence can be classified into three main types: replicative senescence, developmentally programmed cellular senescence, and stress-induced premature senescence [16]. Each stimulus elicits specific phenotypic alterations, underscoring the need for reliable biomarkers, especially robust molecular markers, to accurately identify and characterize senescent cells. Although senescence is generally associated with stable cell-cycle arrest, rare cases indicate that some cells may escape this arrest, suggesting that reversibility, while uncommon, is possible.

A key functional hallmark of senescence is senescence-associated proliferation arrest (SAPA), characterized by persistent cell-cycle arrest. This arrest depends on cyclin-dependent kinase inhibitors such as p16Ink4a (CDKN2A), p27Kip1 (CDKN1B), and p21Waf1 (CDKN1A), which inhibit CDK2 and CDK4/6 activity. The resulting reduction in phosphorylated retinoblastoma protein (p-Rb) decreases transcription of E2F target genes, indicating activation of the p16Ink4a/retinoblastoma (Rb) pathway [27,28]. The transcription of p16 is epigenetically regulated through DNA methylation by DNA (cytosine-5)-methyltransferase 1 and repression by polycomb repressor complexes 1 and 2 at the INK4/ARF locus. During senescence, the inhibition of methyltransferase activity and detachment of these complexes promote euchromatin formation and restore p16 transcription capacity [29,30,31,32].

DNA damage is another central feature of senescence, often elevated due to replication stress and reactive oxygen species that cause DNA breaks and base alterations. Phosphorylation of histone H2AX to γH2AX at Ser139 serves as a critical marker of DNA double-strand breaks and reflects the persistent DNA damage response that is active in senescent cells [33]. Furthermore, senescence is associated with downregulation of SUV39H1, a histone methyltransferase that is important for balancing genomic stability and cell-cycle arrest [34].

**Figure 2:**
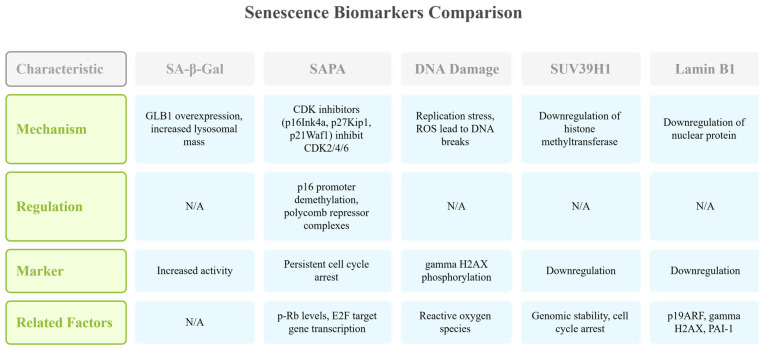
Comparative overview of key senescence biomarkers: This table summarizes major senescence biomarkers, including SA-β-Gal, SAPA, DNA damage, SUV39H1, and Lamin B1. Each marker reflects distinct mechanisms, such as lysosomal expansion, persistent cell-cycle arrest, DNA damage response, and epigenetic or nuclear changes [15,16,26,27,28,29,30,31,32,33,34,35,36,37]. This figure was generated using Napkin AI.

Finally, senescent cells show increased lysosomal mass and protein accumulation, reflected by elevated senescence-associated β-galactosidase (SA-β-Gal) activity due to GLB1 overexpression, making it a widely used early biomarker of senescence [35,36]. Additional markers include downregulation of nuclear lamin B1 protein and increased expression of p19ARF, γH2AX, and plasminogen activator inhibitor-1 (PAI-1), which together characterize the senescent phenotype [37] (Figure 2).

## 3. Senescence Activity on Tumor Cells

### 3.1. Senescence-Involved Signaling Pathways

The initiation of senescence in cancer is driven by several triggers, including DNA damage, telomere shortening, oncogene activation, and various cellular stressors. These factors activate key signaling pathways such as the DNA damage response (DDR), cell-cycle regulation, apoptosis modulation, cellular energy metabolism, and the unfolded protein response (UPR) [38]. A central element of this process is the DDR pathway. Sensor proteins like ATM and ATR detect DNA damage and activate downstream effectors such as CHK1 and CHK2, which halt the cell cycle and initiate DNA repair processes to maintain genomic stability. In senescent cells, persistent DDR activation results in irreversible cell-cycle arrest, thereby preventing the proliferation of damaged cells and serving as a critical tumor-suppressive mechanism that has significant implications for aging and cancer research [39].

Meanwhile, the UPR pathway is triggered by the accumulation of unfolded or misfolded proteins in the endoplasmic reticulum (ER). Sensors including PERK, IRE1, and ATF6 work to restore protein homeostasis by regulating protein synthesis, folding, and degradation. In the context of senescence, UPR activation plays a pivotal role in managing protein quality control and preventing senescence induced by proteotoxic stress [40,41]. Together, these pathways initiate senescence, which can both inhibit and paradoxically contribute to cancer progression.

Lamin A/C is an integral component of the nuclear lamina, providing structural support and regulating various nuclear functions, such as DNA replication, transcription, and chromatin organization, while also interacting with chromatin and binding proteins to regulate cell proliferation and differentiation. Lamin A/C influences several key proteins, including emerin, pRb, c-Fos, SREBP1, and MOK2, and is involved in important signaling pathways such as p53, MAPK, ERK1/2, Wnt, TGF-β, Notch, and NFκB, highlighting its potential as a biomarker for cancer risk [42]. Aberrant localization or misexpression of nuclear lamins is associated with both cancer and accelerated aging. Progerin, an abnormal form of the lamin A protein, is primarily associated with Hutchinson–Gilford Progeria Syndrome (HGPS), a rare genetic disorder characterized by rapid aging. This mutant protein results from defective post-translational processing of prelamin A, leading to defects in nuclear architecture and function. Interestingly, progerin has also been detected in various cancer cells, where it contributes to nuclear abnormalities and influences tumor progression and cellular senescence. The role of progerin in regulating extracellular matrix components, gene expression, and miRNA alterations further emphasizes the complex interplay between cellular structures and senescence in cancer [20,43].

### 3.2. Senescence-Associated Secretory Phenotype

The SASP is a defining feature of senescent cells. It is marked by releasing a wide range of factors, including inflammatory cytokines, chemokines, growth factors, and proteins involved in extracellular matrix remodeling. The SASP plays a dual role in the tumor microenvironment, capable of both suppressing and promoting tumor development [19,44].

Certain SASP factors attract immune cells to remove premalignant senescent cells and support tissue repair. However, other factors can cause chronic inflammation, promote fibrogenesis, and drive tumorigenesis due to ongoing DNA damage signaling [45]. The suppressive function of the SASP includes influencing neighboring stromal, immune, and cancer cells, reducing fibrosis [46,47], aiding wound healing [48,49], and enhancing tissue regeneration [50]. The SASP of senescent cells inhibits cancer’s occurrence and development by inducing and enhancing cell-growth arrest through autocrine or paracrine mechanisms. Key SASP components, such as the interleukins IL-1, IL-6, and IL-8, play crucial roles in this process, with IL-1 being pivotal in activating and maintaining the SASP. Membrane cofactor proteins (MCPs) and macrophage inflammatory proteins (MIPs) are also involved [15,51,52]. The SASP facilitates communication between senescent cells and neighboring cells, exerting antitumor effects. Hence, senescent cells secrete multiple SASP factors, including TGF-β family ligands, CCL2, CCL20, and VEGF, which can damage the DNA of neighboring cells and induce senescence in adjacent cells by increasing the expression of p16, p21, and IL-8 [53,54]. SASP activation of the immune system further aids in cancer cell clearance, as evidenced by the clearance of premalignant senescent hepatocytes through SASP-mediated innate immune activation in mice. This process involves the recruitment of NK cells and M1 macrophages to eliminate senescent or cancer cells [53,55,56].

**Figure 3:**
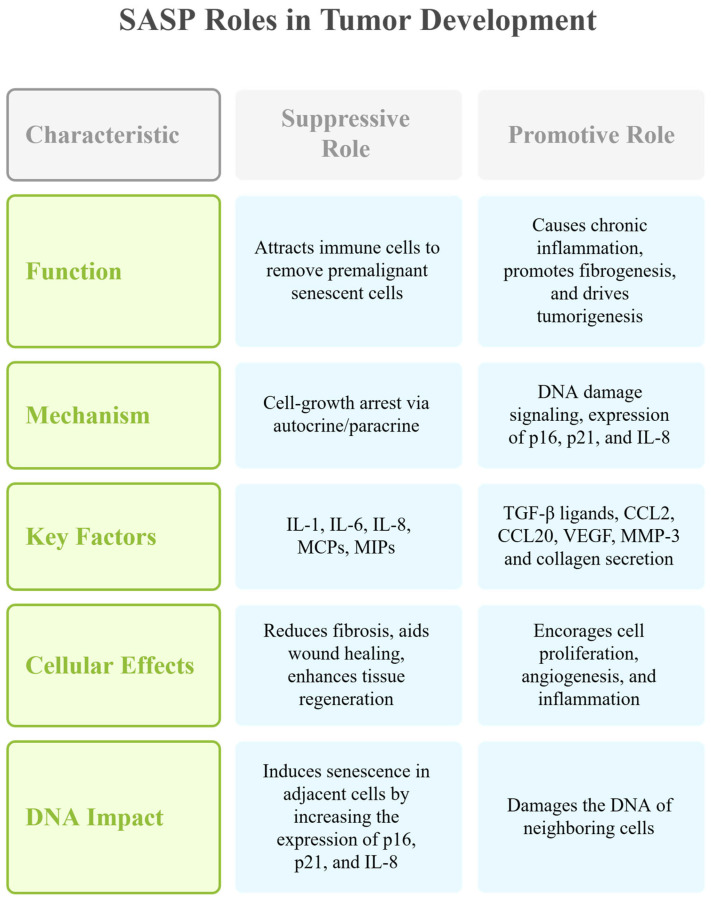
This chart illustrates the paradoxical role of the senescence-associated secretory phenotype in cancer. The SASP can suppress tumor development by enhancing immune-mediated clearance of premalignant cells and promoting tissue repair. Conversely, it can also promote tumorigenesis by inducing chronic inflammation and DNA damage in neighboring cells, and by supporting cancer-related processes like angiogenesis and EMT. Key SASP mediators drive these opposing effects, depending on the cellular context. This figure was generated using Napkin AI.

In specific contexts and with certain cell types, the SASP can facilitate tumor progression by encouraging cell proliferation, angiogenesis, and inflammation [19,57]. For instance, senescent fibroblasts promote the proliferation of preneoplastic epithelial cells by secreting MMP-3 and collagen [15]. This secretion causes epithelial cells to undergo morphological and functional differentiation, thus stimulating tumorigenesis. Additionally, SASP components like IL-6 and IL-8 activate STAT3 signaling, promoting epithelial–mesenchymal transition (EMT)-related gene expression and MMP production, which facilitate cancer cell growth, invasion, metastasis, and tumor vascularization [58]. SASP mediators typically exhibit pro-inflammatory effects, disrupting progenitor and stem cell functions, inducing extracellular matrix rearrangement, and spreading the senescence phenotype. Furthermore, these mediators signal the presence of senescent cells, thereby facilitating their clearance by immune cells (Figure 3).

The complexity of the SASP’s role in oncogenesis likely stems from its intricate regulatory network, which operates at multiple levels. Since cellular senescence is still a relatively poorly understood mechanism, studies indicate that the SASP is regulated by various processes, including chromatin remodeling and the cooperation of specific transcription factors such as NFκB, p53, C/EBP, and GATA4. Additionally, mTOR signaling is crucial in regulating most SASP factors in senescent cells [57]. Components of the trans-Golgi network, such as PRKD1, ARF1, and PI4KIIIβ, and the carrier membrane protein SCAMP4, are upregulated in senescent cells and are necessary for the secretion of many SASP factors [59,60]. Furthermore, genes involved in intracellular trafficking undergo alternative splicing in senescent cells, and PTBP1, a regulator of alternative splicing, is essential for the pro-inflammatory SASP in these cells [61]. Thus, while the SASP exhibits anti-tumorigenic effects, it also has significant pro-tumorigenic properties, underscoring the dual role of cellular senescence in cancer. These are only a few known examples of the SASP’s interaction with diverse regulatory networks, which are likely to expand as the field of cellular senescence evolves.

### 3.3. Senescence Escape and Tumor Aggressiveness

Senescence escape is a critical process contributing to tumor aggressiveness and malignancy, characterized by the ability of cancer cells to circumvent the growth arrest state typically induced by cellular senescence. One of the primary mechanisms by which cancer cells evade this arrest is by reactivating telomerase or utilizing alternative telomere-lengthening mechanisms to maintain telomere integrity and bypass senescence-induced growth arrest [62]. This capability is particularly significant, as telomerase also plays a vital role in aging and longevity by influencing telomere length and regulating processes such as glucose homeostasis and inflammation, key factors in mammalian lifespan [63,64].

Cancer cells that manage to escape senescence acquire distinct characteristics, often correlating with increased tumor aggressiveness. These cells display phenotypic traits such as polyploidy, which enhances the DNA replication potential, along with features of cancer cell stemness marked by biomarkers like CD34 and CD117 [65,66]. As a consequence, senescence-escaped cells are frequently associated with advanced disease stages, increased metastatic potential, and poorer clinical outcomes [67].

The stability of the senescent state is maintained by various mechanisms, including the formation of senescence-associated heterochromatin foci and the trimethylation of histone H3 at lysine 9 (H3K9me3). Alterations in these mechanisms, such as the removal of H3K9me3 by histone demethylases, can facilitate the reversal of senescence, suggesting that inhibiting these demethylases might restore growth arrest in tumor cells [68]. Additionally, the activation of oncogenes and mutations in tumor suppressor genes like TP53 helps cancer cells overcome growth-inhibitory signals, thereby enabling senescence escape and promoting tumor progression [69].

Senescence-escaped cells not only exhibit heightened aggressiveness but also demonstrate substantial resistance to conventional therapies like chemotherapy and radiation, often leading to treatment failure and disease recurrence [70]. Following therapy-induced senescence escape, these cells frequently re-enter the cell cycle with enhanced stem cell-like properties, contributing to cancer recurrence [62]. Furthermore, the senescence-associated secretory phenotype (SASP) plays an important role in this context; the pro-inflammatory environment and extracellular matrix remodeling induced by the SASP can further support cancer cells’ survival and metastatic potential [58,71]. Elucidating these mechanisms is crucial for developing therapeutic strategies that target senescence escape processes, such as telomerase reactivation and epigenetic modifications, ultimately aiming to mitigate the aggressive nature and treatment resistance of cancer cells.

## 4. Senescence-Targeted Cancer Therapies

### 4.1. Oncogene-Induced Senescence and Emerging Therapies Targeting Senescence

Senescence-targeted cancer therapies are an emerging field that leverages the body’s natural cellular aging processes to suppress tumor growth. Cellular senescence, including oncogene-induced senescence (OIS), acts as a crucial barrier against unchecked cancer cell proliferation. Oncogenes like *BRAF*, *AKT*, and *HRASV12*, while driving cell division, also trigger OIS, halting the growth of potentially tumorigenic cells. This dual functionality provides a valuable opportunity to utilize senescence as a therapeutic strategy [15,72,73].

Therapy-induced senescence (TIS) is a promising cancer treatment avenue. This method employs established modalities such as chemotherapy, radiation, immunotherapy, epigenetic modulation, and CDK4/6 inhibitors to induce senescence in cancer cells, thereby stopping their proliferation [74,75]. Notably, TIS also stimulates an immune response against tumor cells and aids in identifying novel therapeutic targets and biomarkers, ultimately aiming to enhance treatment efficacy while minimizing the toxic side effects associated with conventional therapies [8].

Typically, the TIS strategy includes the use of cytotoxic agents such as doxorubicin, etoposide, aphidicolin, bleomycin, cisplatin, mitoxantrone, retinol, and hydroxyurea, which trigger tumor cell-cycle arrest [75]. These drugs can lead to three possible fates—senescence, apoptosis, or necrosis—depending on the administration methods and duration of treatment [76]. Furthermore, etoposide and cisplatin have also been documented to induce senescence in H1299 non-small-cell lung cancer (NSCLC) cells [77], while the BET inhibitors JQ1 and iBET762 triggered senescence in squamous-cell carcinomas and mucoepidermoid carcinomas from the head and neck area [78,79]. CDK4/6 inhibitors such as palbociclib have proven effective in inducing senescence in breast cancer cells, among others, by interfering with the G1-to-S phase transition of the cell cycle. Palbociclib has been demonstrated to reduce tumor volume and, in some cases, activate immune surveillance mechanisms [80,81]. Additionally, variants like ribociclib and abemaciclib have been approved for use in hormone-receptor-positive breast cancers, highlighting the clinical utility of these inhibitors [82,83]. Palbociclib has successfully induced cellular senescence in melanoma, liposarcoma, gastric cancer, and hepatocellular carcinoma by inhibiting the mammalian target of rapamycin (mTOR) signaling pathway [84,85].

Overall, senescence-targeted therapies offer a promising route to suppress tumor growth and improve therapeutic outcomes, especially in apoptosis-resistant cancers. However, careful consideration is needed to balance the beneficial effects of induced senescence with potential adverse effects, including the induction of senescence in non-cancerous cells and subsequent long-term side effects [15,86].

### 4.2. Targeting Senescence in Cancer—Senotherapies

Targeting senescence in cancer through senotherapies is emerging as a promising strategy. Senotherapies encompass senolytic drugs, which selectively eliminate senescent cells; senostatic drugs, which inhibit their function; and senomorphic drugs, which regulate their activity without eliminating them [87].

Senolytics focus on targeting anti-apoptotic pathways that are overexpressed in cancer cells, such as the BCL-2 family and FOXO4-p53 interaction, with agents like HDAC inhibitors (panobinostat), BCL-2 inhibitors (ABT-737, ABT-263), and natural compounds (quercetin, navitoclax, fisetin). These drugs disrupt survival pathways that are crucial to the maintenance of senescent cells, enhancing the effectiveness of cancer therapies and preventing tumor progression [88,89]. For instance, dasatinib with quercetin has reduced senescent cell burdens in aged individuals and conditions like diabetic kidney disease [90]. Additionally, MEK inhibitors such as trametinib have shown efficacy in inducing oncogene-induced senescence in certain cancers by targeting the MEK1/2 pathway [91].

Senomorphics, which target key senescent cell pathways like p38 MAPK, NFκB, and mTOR, suppress the SASP without causing cell death. Agents like rapamycin, metformin, apigenin, and kaempferol have shown promise in reducing the SASP and exerting tumor-suppressive effects [92,93].

## 5. Senescence and Head and Neck Cancers

Head and neck cancers (HNCs) refer to a diverse group of cancers originating in the upper aerodigestive tract, including regions such as the oral cavity, pharynx, larynx, nasal passages, salivary glands, and thyroid. While there have been advances in identifying biomarkers and developing new therapeutics, these improvements have not substantially increased the overall survival rates. This is largely due to the persistently high rates of local recurrence and metastasis associated with the disease. The complexity of these tumors and their aggressive nature highlight the need for more effective treatment strategies and comprehensive approaches to early detection and personalized medicine to improve outcomes for patients with HNSCC.

### Emerging Opportunities for Senescence-Induced Therapies

Emerging therapeutic approaches in head and neck squamous-cell carcinoma (HNSCC) increasingly focus on manipulating the interactions between tumor cells and the immune system, which often employ immunosuppressive mechanisms such as immune checkpoint expression and recruitment of regulatory T cells and myeloid-derived suppressor cells (MDSCs) [67,70]. Preclinical studies have demonstrated that combining senescence-inducing agents with conventional treatments can enhance antitumor efficacy. For example, cytotoxic agents like cisplatin induce senescence in HNSCC cells and show some senolytic potential by targeting growth-arrested cancer cells. However, their senolytic effectiveness is relatively limited compared to more specialized agents [89,94]. Sequential administration of cisplatin followed by navitoclax, a BCL-2/BCL-XL inhibitor, has shown promising preclinical results by selectively inducing apoptosis in senescent cells, thus potentially reducing the risk of cancer recurrence [89,94]. This preclinical evidence highlights the benefit of combining senescence induction with targeted clearance of senescent cells, but clinical translation remains under evaluation.

Oxidative stress is a major contributor to cellular senescence and significantly impacts head and neck cancers, where tumor cells often upregulate antioxidant enzymes such as paraoxonase-2 to counteract high levels of reactive oxygen species (ROS) and develop resistance to treatments like cisplatin [95,96,97,98,99,100]. Targeting these antioxidant defenses, including metabolic enzymes like ME2, can disrupt ROS homeostasis, leading to increased oxidative damage and promoting therapy-induced senescence. In particular, ME2 depletion raises ROS levels and sensitizes p53-mutant HNSCC cells to senescence when combined with treatments like metformin, which alone has limited senescence-inducing effects. This strategy may enhance the efficacy of conventional therapies such as radiation and cisplatin by pushing ROS beyond harmful thresholds. Notably, this mechanism appears to function independently of p53 status and AMPK signaling, suggesting the involvement of alternative ROS-related pathways [101].

Senescence escape is increasingly recognized as a key driver of tumor aggressiveness and therapy resistance in head and neck squamous-cell carcinoma (HNSCC). Functional studies in HNSCC cell lines treated with cisplatin demonstrate that cells initially driven into senescence can later resume proliferation, lose senescence-associated markers, and reacquire tumor-initiating potential, hallmarks of senescence reversal [94]. This process is tightly linked to the molecular and immunological heterogeneity of HNSCC. Certain tumors present with elevated CDKN2A expression, reduced SASP activity, poor immune infiltration, and frequent TP53 mutations, indicating a microenvironment that favors the persistence and eventual escape of senescent cells [102,103]. Conversely, tumors with stronger immune activation show more effective clearance of senescent cells and are associated with better prognosis [104]. Together, these findings suggest that failure to eliminate senescent cells promotes their escape and enhances malignant traits, reinforcing the need for biomarkers and therapies that can identify and eliminate senescence-escaped cells to prevent recurrence and progression.

Navitoclax, in particular, has demonstrated efficacy in preclinical HNSCC models by effectively eliminating cisplatin-induced senescent cells, preventing tumor regrowth, and improving survival outcomes. Despite these encouraging findings, clinical use of navitoclax and other BCL-2 family inhibitors faces significant challenges due to toxicity concerns, notably thrombocytopenia caused by BCL-XL inhibition in platelets [105,106]. This toxicity limits dosing and treatment duration in patients. Nonetheless, robust clinical trial data in HNSCC specifically remain limited, underscoring the need for further studies to evaluate the safety and efficacy of these agents in patients.

In addition to navitoclax, CDK4/6 inhibitors like palbociclib have been shown to induce senescence through the CDK4/6-Rb pathway, particularly in HPV-negative HNSCC [107]. When combined with navitoclax, this dual approach enhances the selective clearance of senescent cells, potentially overcoming therapeutic resistance. Furthermore, combination therapies pairing CDK4/6 inhibitors with PI3K inhibitors such as alpelisib have demonstrated synergistic effects, especially in tumors harboring PIK3CA mutations [108]. While these combination strategies show promise in preclinical models, their translation into clinical protocols requires careful assessment of cumulative toxicities and patient tolerance.

The involvement of bromodomain proteins, especially BRD4, has been reported in the progression and therapeutic resistance of squamous-cell carcinoma from the head and neck and mucoepidermoid carcinoma from the salivary glands. Bromodomain proteins are epigenome readers that modulate gene transcription through interactions with histone tails, implicating them in oncogene regulation and inflammatory processes in these cancers. In mucoepidermoid carcinoma, overexpression of BRD4 has been linked to tumorigenicity, with research demonstrating that using the BET inhibitor iBET762 to displace BRD4 from chromatin leads to cell-cycle arrest and activation of cellular senescence, as indicated by increased levels of p16ink4. This approach also depletes cancer stem cell populations, hinting at a novel therapeutic strategy that disrupts epigenetic deregulation. Similarly, in HNSCC, the bromodomain inhibitor JQ1 disrupts BRD4’s interaction with histones, leading to cellular senescence characterized by DNA damage and loss of cancer stem cells [78,80]. Furthermore, the use of epigenetic modulators, such as HDAC inhibitors like panobinostat and vorinostat, can amplify senolytic effects by activating tumor suppressor genes and promoting senescence in head and neck squamous-cell carcinoma and adenoid cystic carcinomas from the salivary glands [44,89].

Another critical regulator of cellular senescence that warrants attention is Sirt1, an NAD+-dependent deacetylase. Nicotinamide, a substrate of nicotinamide N-methyltransferase (NNMT), is also a potent inhibitor of Sirt1. NNMT is upregulated in various solid tumors, including head and neck cancers, where it contributes to tumor aggressiveness [109]. Elevated NNMT activity lowers intracellular nicotinamide levels through methylation, thereby maintaining Sirt1 activity and potentially suppressing senescence. Blocking NNMT could increase nicotinamide availability, inhibit Sirt1, and promote senescence, leading to tumor growth suppression. Several NNMT inhibitors have been proposed for translational applications, including macrocyclic peptides functioning as allosteric inhibitors, alkene-linked bisubstrate inhibitors, and esterase-sensitive prodrugs of bisubstrate inhibitors [109]. These agents represent promising avenues to induce senescence in head and neck tumors, particularly when conventional treatments fail.

Leveraging cellular senescence as a stratification tool in HNSCC offers a promising new avenue for differentiating prognostic subgroups in these highly heterogeneous tumors. Given that head and neck squamous-cell carcinoma exhibits significant molecular and clinical variability, traditional treatment approaches often fall short of achieving personalized care. Identifying senescent tumor cells through robust biomarkers, such as the cyclin-dependent kinase inhibitors p16^INK4A^ and p21^CIP1^, along with SASP indicators characterized by elevated interleukins (IL-6, IL-8) and chemokines like CXCL1 [102,110,111], provides critical insights into the underlying biology of these cancers. More recently, the discovery of specific gene signatures (e.g., *BTG3*, *EHF*, *EZH2*, *TACC3*, *TXN*) and senescence-related long non-coding RNAs [102,104] has further refined our ability to gauge senescent states. Together, these biomarkers not only reflect key aspects of the tumor microenvironment, such as immune cell infiltration, extracellular matrix remodeling, and mutational burden, but also lay the groundwork for developing robust scoring systems aimed at patient stratification in HNSCC.

**Figure 4:**
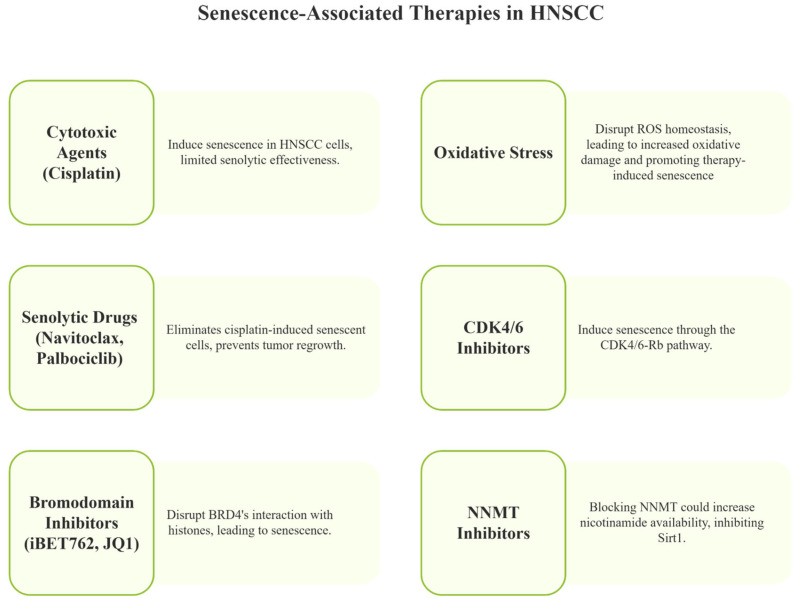
This diagram outlines therapies that induce or enhance senescence in head and neck cancer. Approaches include cytotoxic agents, senolytic drugs, oxidative stress modulation, CDK4/6 and bromodomain inhibitors, and NNMT inhibition. Together, these strategies aim to suppress tumor growth by promoting or clearing senescent cancer cells. This figure was generated using Napkin AI.

Therefore, preclinical evidence supports senescence-targeted therapies in HNSCC, but clinical translation faces challenges such as toxicity from senolytics, especially BCL-2/BCL-XL inhibitors, and a lack of predictive biomarkers. Future trials should focus on safety, combination treatments, and biomarker integration to optimize patient outcomes. More mechanistic research and clinical validation are needed to fully realize the therapeutic potential of senescence modulation in HNSCC (Figure 4).

## 6. Future Perspectives

Harnessing cellular senescence as a therapeutic strategy in cancers, including HNSCC, is gaining momentum due to growing insights into senescence’s dual role in tumor progression and resistance. Senescent cells initially halt proliferation and suppress tumor growth [15,16], yet if not effectively cleared they may contribute to relapse and metastasis through the senescence-associated secretory phenotype (SASP) [8,19]. The complexity of senescence regulation, involving intricate signaling networks, presents both challenges and opportunities for therapy development. Preclinical studies have demonstrated that combining senescence induction with selective clearance of senescent cells can reduce tumor recurrence and improve survival [89,94]. However, toxicity issues limit clinical use, necessitating the development of safer, more selective senolytics [105,106].

Combining senescence-inducing therapies with immune-modulating agents represents a promising future direction. The immune system plays a critical role in clearing senescent cells, and boosting the activity of natural killer (NK) cells and macrophages could enhance the efficacy of senescence-based treatments [112,113]. Additionally, targeting oxidative stress pathways by disrupting antioxidant defenses, such as paraoxonase-2 and metabolic enzymes like ME2, may sensitize tumor cells to therapy-induced senescence even in p53-mutant HNSCC, offering a strategy independent of canonical pathways [101]. Epigenetic regulators, including bromodomain proteins (e.g., BRD4) and enzymes like Sirt1, also emerge as important targets. BET inhibitors (JQ1, iBET762) and HDAC inhibitors (panobinostat, vorinostat) can promote senescence and diminish cancer stem cell populations in HNSCC and related carcinomas [44,78,79,89]. Furthermore, inhibiting nicotinamide N-methyltransferase (NNMT) to suppress Sirt1 activity is an additional avenue to induce senescence in these tumors [109].

Despite these advances, several significant challenges remain. Tumor heterogeneity leads to variable senescence responses; not all cancer cells uniformly enter senescence, and some senescent cells may acquire stem cell-like features, complicating treatment [8]. Developing reliable biomarkers to identify and monitor senescent cells in clinical settings is critical for optimizing patient selection and treatment response [62]. Moreover, resistance to senolytic agents can limit efficacy when single agents fail to target all senescent cell subtypes. Exploring combination therapies that address multiple survival pathways is essential to overcome this obstacle. Current research in HNSCC is uncovering novel targets to enhance sensitivity to senolytics, expanding the therapeutic options for patients [93,101].

In summary, the future of senescence-based cancer therapy, particularly in HNSCC, will likely depend on multi-modal approaches that integrate senescence induction with selective senolytic clearance, immune modulation, and targeted interventions. Optimization of combination regimens, alongside biomarker-guided patient stratification, will be crucial to improving efficacy and minimizing toxicity. Continued mechanistic studies and well-designed clinical trials are necessary to fully realize the therapeutic potential of manipulating cellular senescence to enhance precision and outcomes in cancer care.

## 7. Conclusions

This review highlights the multifaceted nature of senescence, delineating its dual influence on tumor suppression and aggressiveness, and emphasizes the importance of context-dependent regulation. Advances in senescence-inducing therapies, combined with targeted senolytic strategies and immune modulation, offer promising avenues to enhance therapeutic efficacy and reduce recurrence. Nevertheless, the heterogeneous response of tumors to senescence, along with challenges in identifying specific biomarkers and overcoming therapeutic resistance, remains a significant barrier. Major obstacles preventing translation into clinical practice include the complexity of senescence signaling networks, variability in senescent cell clearance, toxicity concerns with senolytic agents, and the lack of reliable biomarkers to monitor therapy. Future directions should prioritize the development of combination treatments that integrate senescence modulation with precise molecular targeting and immune system engagement, alongside robust biomarker discovery and strategies to mitigate resistance and side effects.

## Declaration of use of Generative AI

To enhance the readability and grammatical accuracy of this manuscript, we utilized the UM-GPT tool, an AI technology developed by the University of Michigan. This tool assisted in proofreading the text, ensuring clarity and coherence. All substantive content, data analysis, and conclusions drawn within this manuscript are the result of the authors’ original work and have not been altered by the AI tool. All figures were generated using Napkin AI, which was employed to help visually summarize complex concepts and organize key ideas in a concise and accessible format.
